# Supplementary figures and images for: Binding mechanism of oseltamivir and influenza neuraminidase suggests perspectives for the design of new anti-influenza drugs
Source: PLoS Comput Biol. 2022 Jul 28;18(7):e1010343. doi: 10.1371/journal.pcbi.1010343 (PMC9401145; doi:10.1371/journal.pcbi.1010343)

S0A

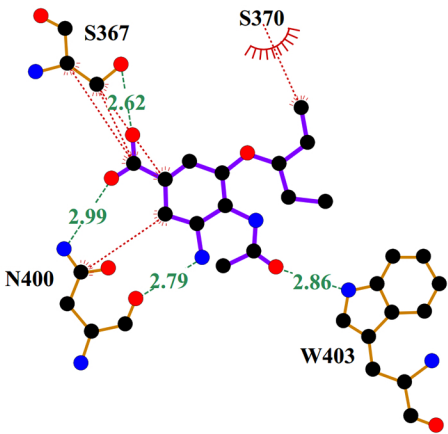

S0B

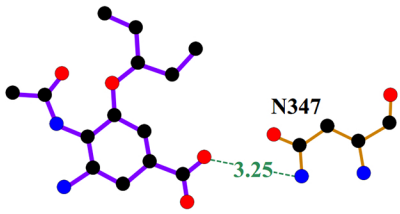

S5

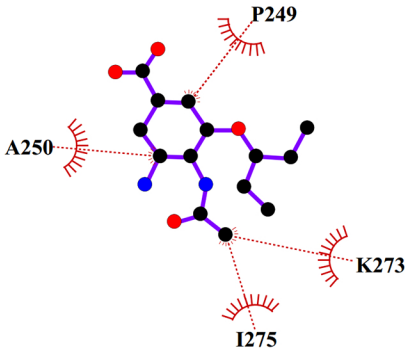

S6

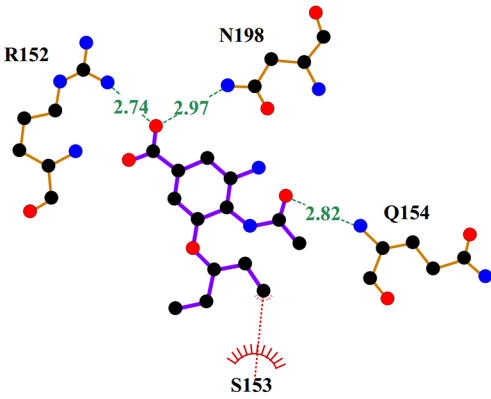

S2

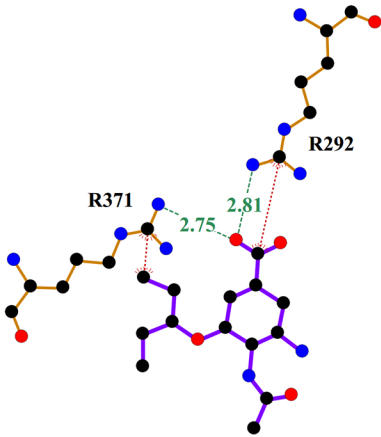

S3

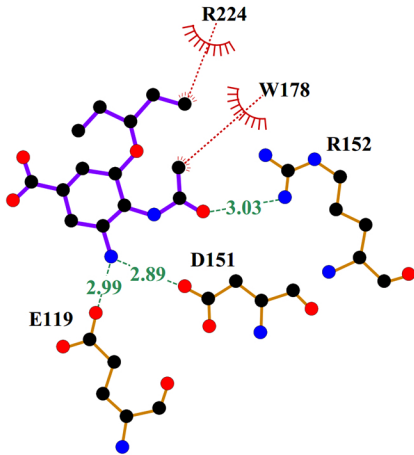

S4

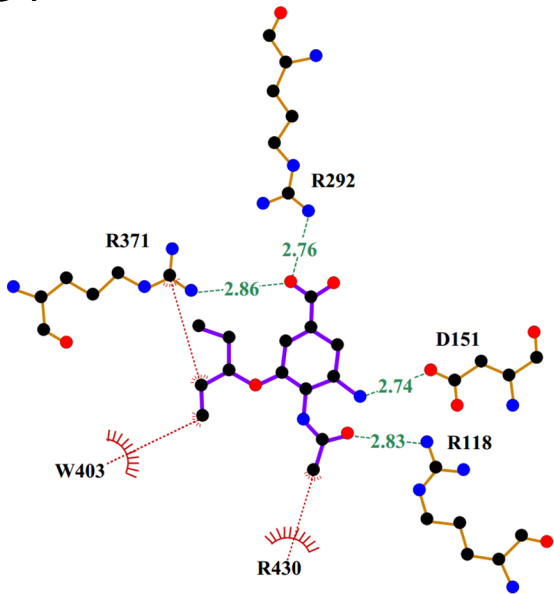

S7

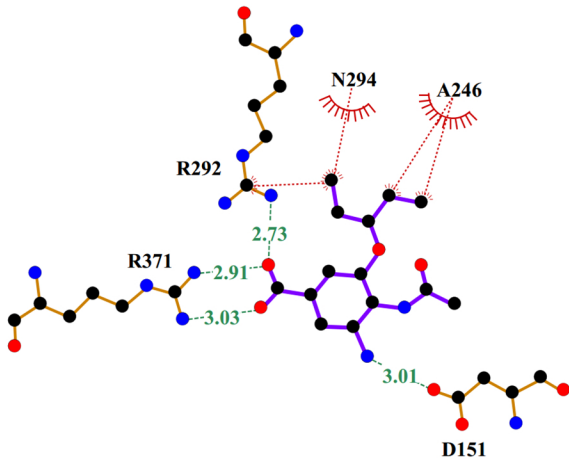

S8

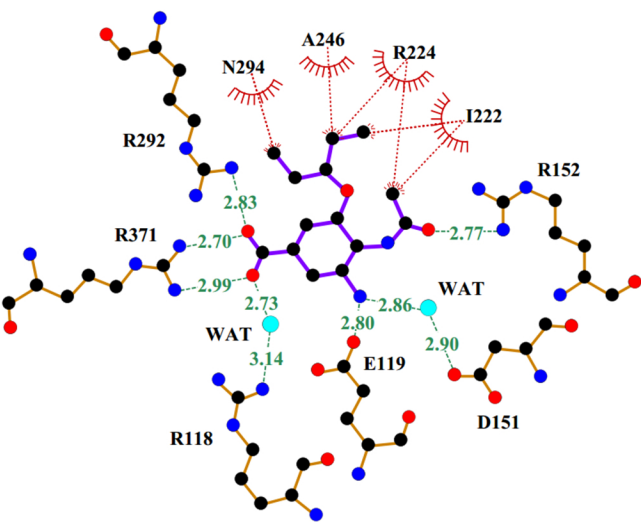

S9

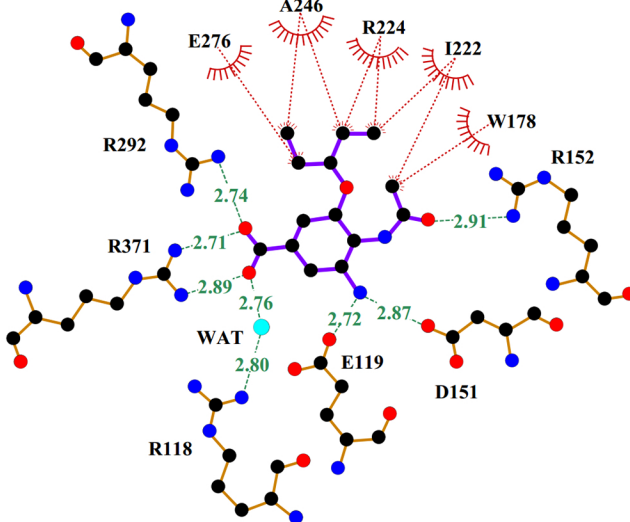

Supplement: S1 Fig — Ligplot+ was used to plot the interaction between oseltamivir and NA in metastable states. Hydrogen bonds are shown as green dotted lines. Hydrophobic contacts are shown as red dotted lines. (PDF) [file pcbi.1010343.s001.pdf]

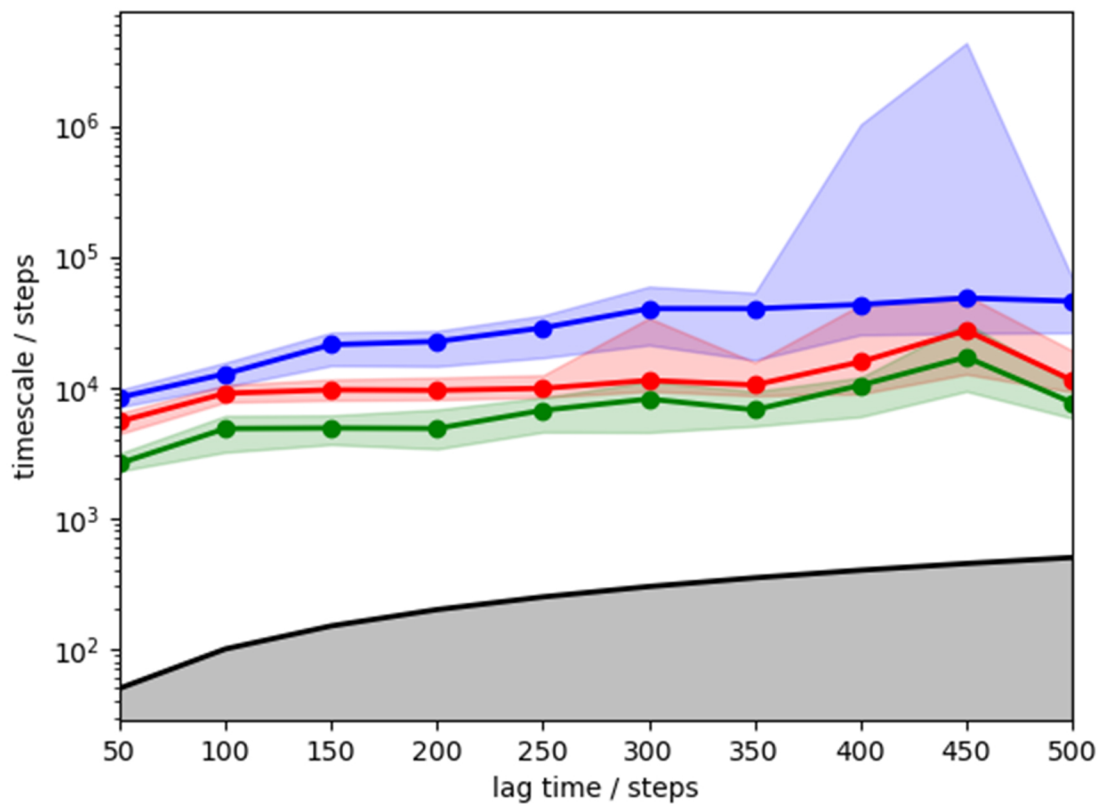

Supplement: S2 Fig — Implied time scale plots with errors as a function of lag time for the HMMs constructed based on the simulation data. Each colored line represents the timescales of different dynamical processes (motions) identified by the decomposition of the transition matrix (eigenvalues). If the model was Markovian (at the chosen lag time), then the timescales would be constant for all longer lag times that were also short enough to resolve the process. Bayesian errors are indicated as similarly colored shaded areas. (PDF) [file pcbi.1010343.s002.pdf]

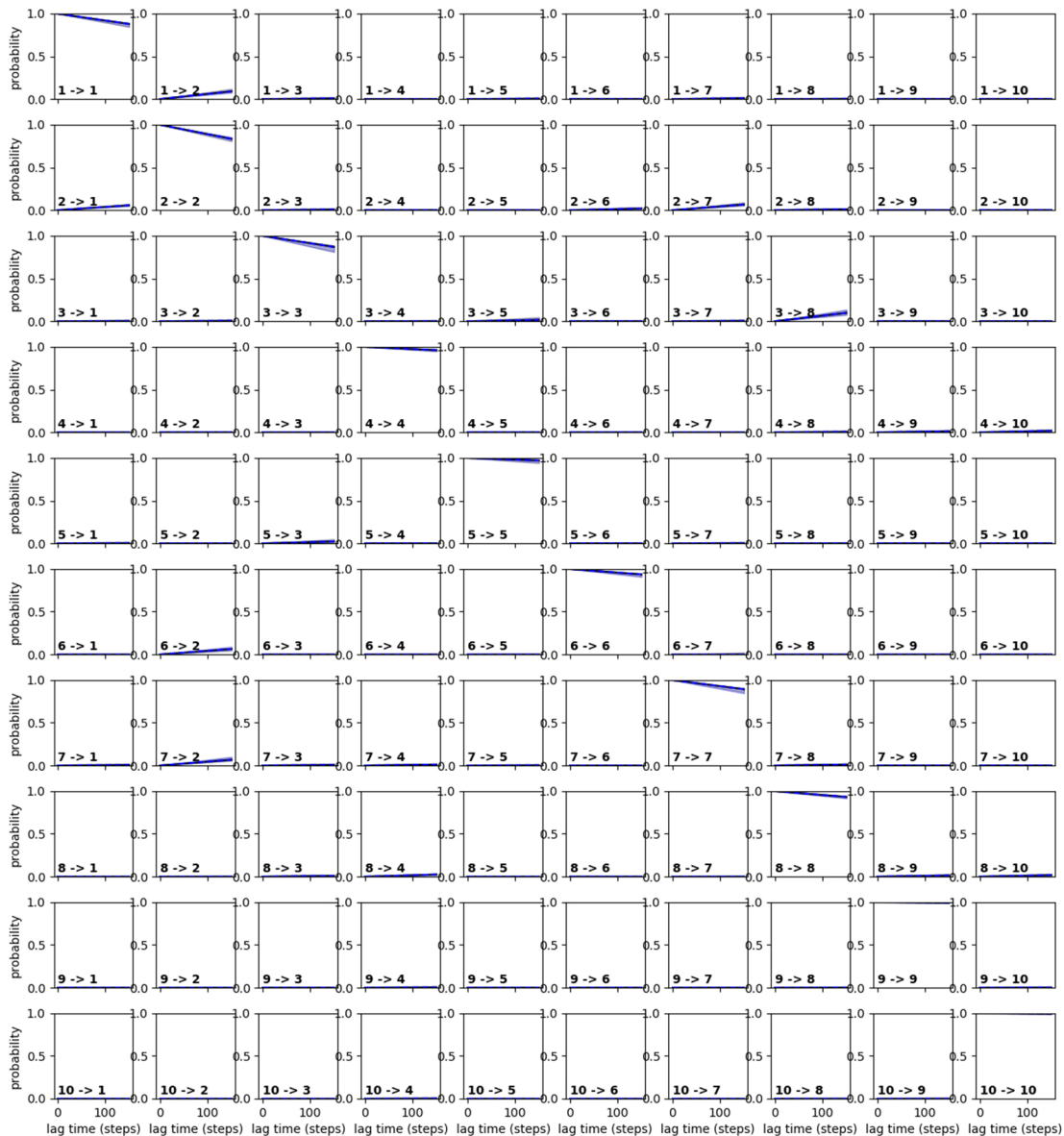

Supplement: S3 Fig — The plots indicate convergence of the presented models. (PDF) [file pcbi.1010343.s003.pdf]

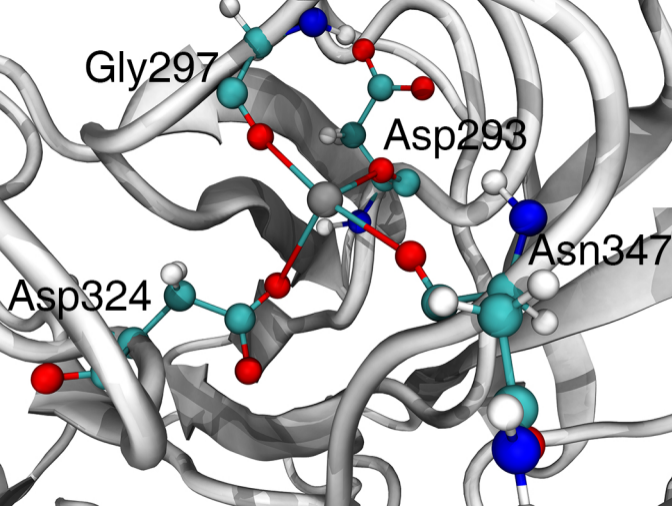

Supplement: S4 Fig — (PDF) [file pcbi.1010343.s004.pdf]

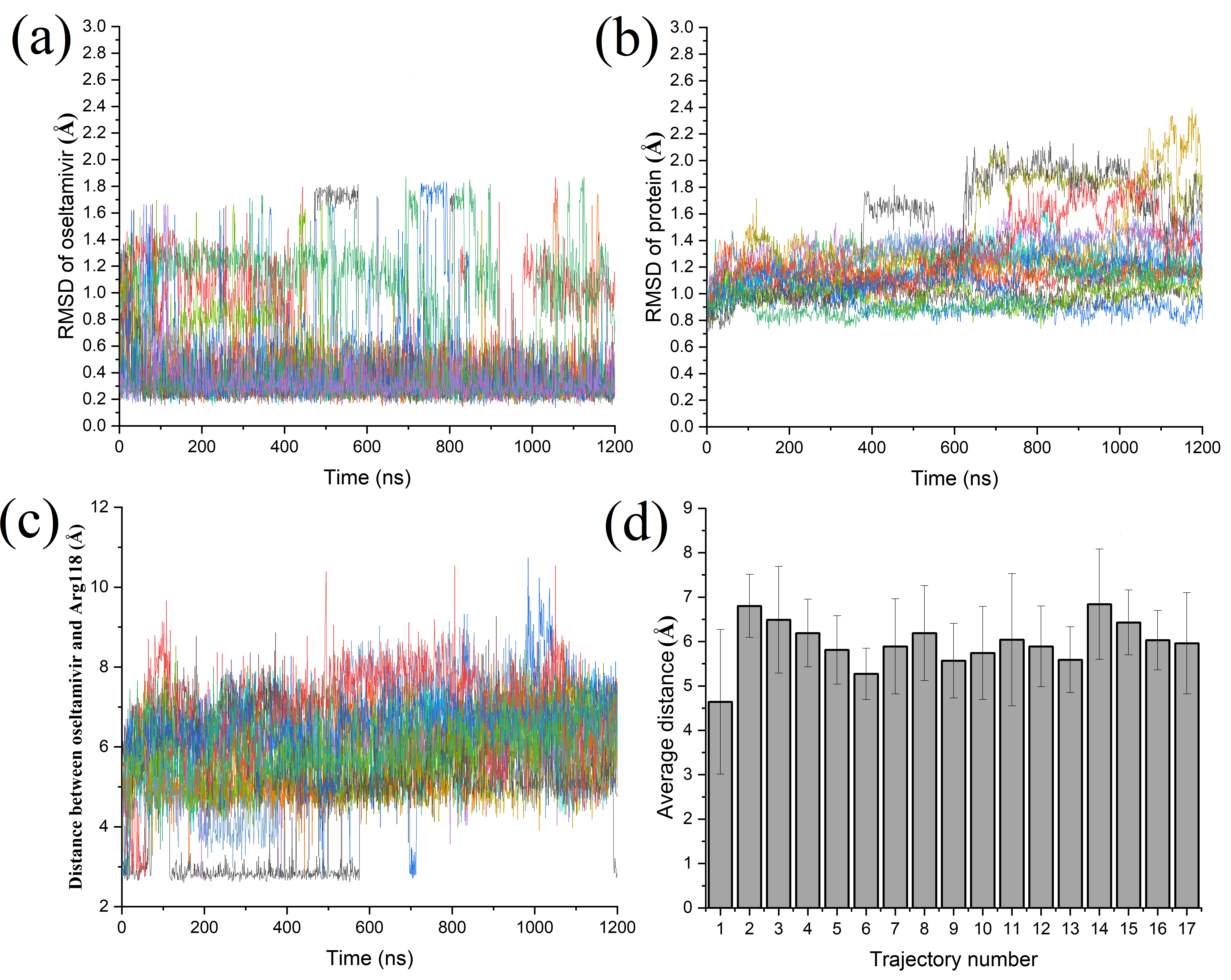

Supplement: S5 Fig — The simulations were performed with oseltamivir-NA complex of NA tetramer starting from the crystal structure (PDB ID: 2QWK). (a) The RMSD values of oseltamivir and (b) NA. (c) Time evolution of distance between the O1B atom of oseltamivir and NH1 atom of Arg118 in the 16 individual oseltamivir-NA complex. (d) Column 1–16 is the average distance between the O1B atom of oseltamivir and NH1 atom of Arg118 of individual oseltamivir-NA complex (4.64 Å ~ 6.84 Å), column 17 is the average distance between the O1B atom of oseltamivir and NH1 atom of Arg118 of all oseltamivir-NA complex (5.96Å). (TIF) [file pcbi.1010343.s005.tif]

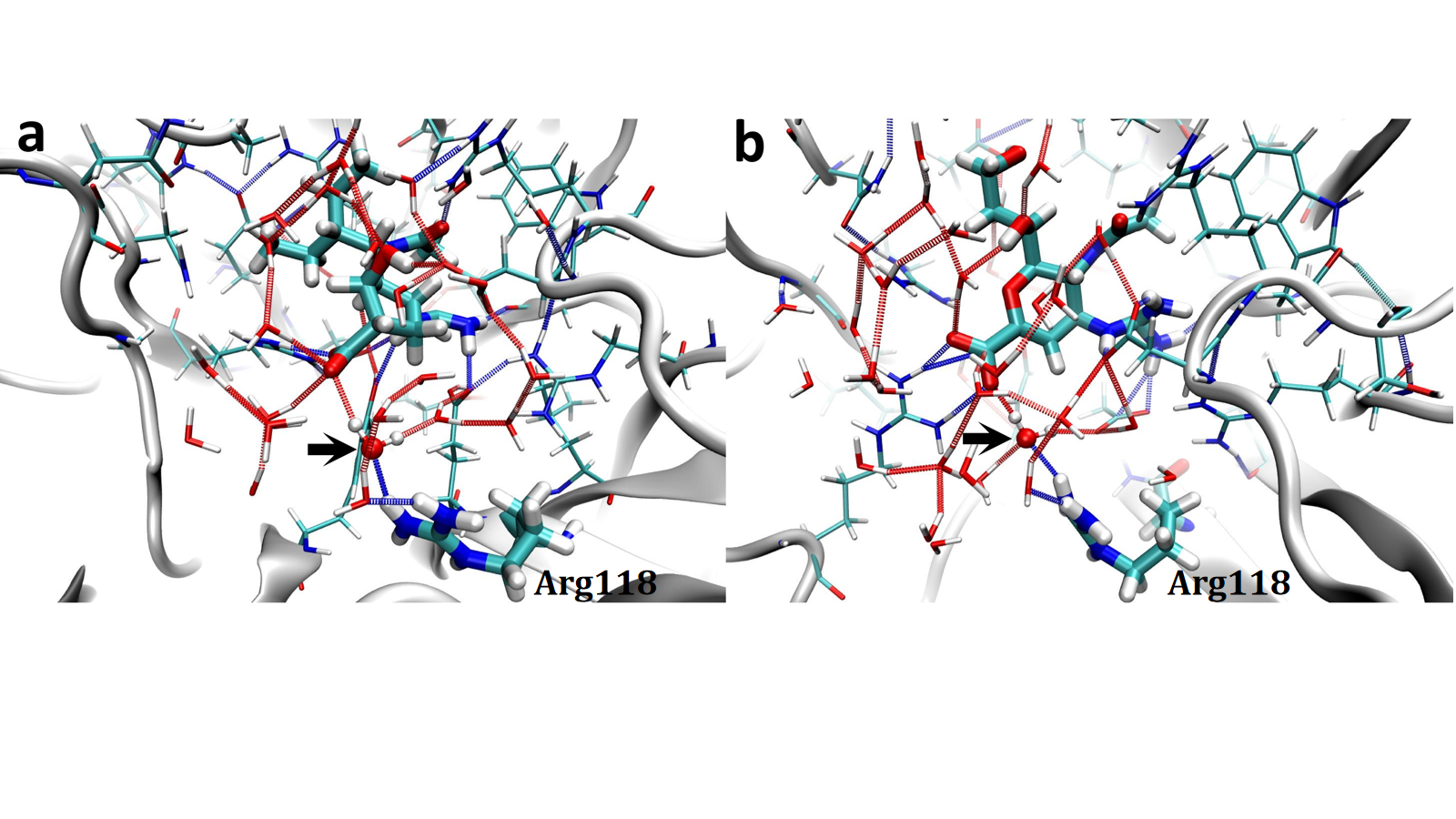

Supplement: S6 Fig — (TIF) [file pcbi.1010343.s006.tif]
