# Supplementary material for: Binding mechanism of oseltamivir and influenza neuraminidase suggests perspectives for the design of new anti-influenza drugs
Source: PLoS Comput Biol. 2022 Jul 28;18(7):e1010343. doi: 10.1371/journal.pcbi.1010343 (PMC9401145; doi:10.1371/journal.pcbi.1010343)
Supplement: S1 Appendix — Section 1: Detailed parameters for molecular dynamics (MD) simulations. Section 2: Detailed parameters of the meta-eABF simulation. Section 3: Analysis of the H-bond between the carboxylate group of oseltamivir and the guanidine group in the crystal structure containing oseltamivir and influenza NA. Section 4: Transition path theory fluxes from the unbound state (S1) to the bound state (S9). Section 5: Pathway analysis based on metastable states. Section 6: Mean first passage time between metastable states. Section 7: Comparison of residue numbers of NA between our simulation system and the PDB 2QWK crystal structure. Table A. Partial Charge and Atom Type of the Metal Center. Table B. Basic information about the oseltamivir-NA crystal structure. Table C. Net flux (×10−6/ns) from the unbound state (S1) to the bound state (S9). Table D. Dominant reaction pathways between the unbound state (S1) and the bound state (S9). Table E. Mean first passage time between metastable states. Table F. Comparison of residue numbers of NA between our simulation system and the crystal structure PDB 2QWK. (DOCX) [file pcbi.1010343.s007.docx]

**Supplementary Information for**

**Binding mechanism of oseltamivir and influenza neuraminidase suggests perspectives for the design of new anti-influenza drugs**

Jiaye Tao^1☯^, Heping Wang^2☯^, Wenjian Wang^☯^, Na Mi^1^, Wei Zhang^1^, Qiujia Wen^1^, Jiajing Ouyang^1^, Xinyun Liang^1^, Min Chen^1^, Wentao Guo^1^, Guoming Li^1^, Jun Liu^1^, Hanning Zhao^1^, Xin Wang^1^, Xuemeng Li^1^, Shengjun Feng^1^, Xinguang Liu^3^*, Zhiwei He^4^* Zuguo Zhao^1^*

**^1^** Department of Microbiology and Immunology of Basical Medicine of Guangdong Medical University, No. 1 Xincheng Road, Dongguan City, 523808, Guangdong Province, China.

**^2^** Department of Respiratory diseases of Shenzhen Children’s Hospital, No.7019 Yitian Road, Futian district, Shenzhen, 518038, Guangdong, China.

**^3^** Guangdong Provincial Key Laboratory of Medical Molecular Diagnostics, Institute of Aging Research,Guangdong Medical University, Dongguan, China.

**^4^** Department of Sino US joint Cancer Institute of Guangdong Medical University, No. 1 Xincheng Road, Dongguan City, 523808, Guangdong Province, China.

^☯^,These authors contributed equally to this work.

*Xingguang Liu, [xgliu@gdmu.edu.cn](mailto:xgliu@gdmu.edu.cn)

*Zhiwei He, [hezhiwei@gdmu.edu.cn](mailto:hezhiwei@gdmu.edu.cn)

*Zuguo Zhao, [zhaozuguo@gdmu.edu.cn](mailto:zhaozuguo@gdmu.edu.cn)

**Contents**

**Section 1:** Detailed parameters for molecular dynamics (MD) simulations

**Section 2:** Detailed parameters of the meta-eABF simulation

**Section 3:** Analysis of the H-bond between the carboxylate group of oseltamivir and the guanidine group in the crystal structure containing oseltamivir and influenza NA

**Section 4:** Transition path theory fluxes from the unbound state (S1) to the bound state (S9)

**Section 5:** Pathway analysis based on metastable states

bound state (S9)

**Section 6:** Mean first passage time between metastable states

**Section 7:** [Comparison](https://cn.bing.com/dict/search?q=comparisons&FORM=BDVSP6&cc=cn) [of](https://cn.bing.com/dict/search?q=of&FORM=BDVSP6&cc=cn) residue numbers of NA between our simulation system and the PDB 2QWK crystal structure

**Table legends in SI Appendix**

**Table A. Partial Charge and Atom Type of the Metal Center**

**Table B. Basic information about the oseltamivir-NA crystal structure**

**Table C. Net flux (×10^-6^/ns) from the unbound state (S1) to the bound state (S9)**

**Table D. Dominant reaction pathways between the unbound state (S1) and the**

**Table E. Mean first passage time between metastable states**

**Table F.** [**Comparison**](https://cn.bing.com/dict/search?q=comparisons&FORM=BDVSP6&cc=cn) [**of**](https://cn.bing.com/dict/search?q=of&FORM=BDVSP6&cc=cn)**residue numbers of NA between our simulation system and the crystal structure PDB 2QWK**

**Section 1: Detailed parameters for molecular dynamics (MD) simulations**

**A. System for the free-binding MD simulation with a single oseltamivir molecule and an NA monomer.** The system for the free-binding MD simulation was a truncated octahedral box containing 14184 water molecules, 52 sodium ions, 55 chloride ions and one oseltamivir molecule (a total of 48674 atoms). The thickness of the water layer was 15 Å.

**B. System for the free-binding MD simulation with four oseltamivir molecules and an NA tetramer.** The system was a truncated octahedral box containing 47160 water molecules, 130 sodium ions, 142 chloride ions and four oseltamivir molecules (a total of 165812 atoms). The diffusion space of each oseltamivir molecule was restricted to an individual NA monomer, as described in the main text.

**C. Parameters for the bonded model of Ca^2+^ and coordinated residues (Table A).** The residues coordinated to calcium ions were D293, G297, D324, and N347 in PDB 2QWK (S4 Fig). The same bonded-model parameters were used for the simulation with a monomer or a tetramer.

The detailed parameters for the bonded model were as follows:

**Table A. Partial Charge and Atom Type of the Metal Center**

| Residue | Atom name | Atom type | Charge | Atom name | Atom type | Charge |
| --- | --- | --- | --- | --- | --- | --- |
| AP1 (D293) | N | N | -0.443248 | HB3 | HC | 0.014458 |
|  |  |  |  |  |  |  |
|  | H | H | 0.283149 | CG | CO | 0.478355 |
|  | CA | CX | 0.088091 | OD1 | O2 | -0.520893 |
|  | HA | H1 | 0.100914 | OD2 | O2 | -0.602844 |
|  | CB | 2C | 0.003842 | C | C | 0.451895 |
|  | HB2 | HC | 0.014458 | O | Y1 | -0.65357 |
| GY1 (G297) | N | N | -0.129921 | HA3 | H1 | 0.215388 |
|  |  |  |  |  |  |  |
|  | H | H | 0.203963 | C | C | 0.83393 |
|  | CA | CX | -0.574338 | O | Y2 | -0.630203 |
|  | HA2 | H1 | 0.215388 |  |  |  |
| AP2 (D324) | N | N | -0.5163 | HB3 | HC | -0.102316 |
|  |  |  |  |  |  |  |
|  | H | H | 0.309869 | CG | CO | 0.586906 |
|  | CA | CX | 0.0381 | OD1 | O2 | -0.570755 |
|  | HA | H1 | 0.110071 | OD2 | Y3 | -0.700083 |
|  | CB | 2C | 0.167085 | C | C | 0.5366 |
|  | HB2 | HC | -0.102316 | O | O | -0.5819 |
| AN1 (N347) | N | N | 0.422476 | CG | C | 0.613325 |
|  | H | H | 0.16316 | OD1 | O | -0.515674 |
|  | CA | CX | -0.073547 | ND2 | N | -0.817566 |
|  | HA | H1 | 0.110329 | HD21 | H | 0.379117 |
|  | CB | 2C | -0.008544 | HD22 | H | 0.394604 |
|  | HB2 | HC | 0.022996 | C | C | 0.645874 |
|  | HB3 | HC | 0.022996 | O | Y4 | -0.5664 |
| CA1 (Ca2+ 999) | CA | M1 | 1.528031 |  |  |  |

**Additional force field parameters for the metal center.**

**MASS**

M1 40.08 Ca ion

Y1 16.00 0.434 carbonyl group oxygen

Y2 16.00 0.434 carbonyl group oxygen

Y3 16.00 0.434 carboxyl and phosphate group oxygen

Y4 16.00 0.434 carbonyl group oxygen

**BOND**

Y1-M1 25.7 2.4095 Created by the Seminario method using MCPB.py

Y2-M1 25.6 2.3724 Created by the Seminario method using MCPB.py

Y3-M1 20.6 2.4724 Created by the Seminario method using MCPB.py

Y4-M1 26.5 2.3689 Created by the Seminario method using MCPB.py

C -Y1 570.0 1.229 JCC,7,(1986),230; AA,CYT,GUA,THY,URA

C -Y2 570.0 1.229 JCC,7,(1986),230; AA,CYT,GUA,THY,URA

C -Y4 570.0 1.229 JCC,7,(1986),230; AA,CYT,GUA,THY,URA

CO-Y3 656.0 1.2500

**ANGL**

C -Y1-M1 25.73 128.02 Created by the Seminario method using MCPB.py

C -Y2-M1 30.75 146.32 Created by the Seminario method using MCPB.py

C -Y4-M1 30.97 146.89 Created by the Seminario method using MCPB.py

CO-Y3-M1 75.91 92.00 Created by the Seminario method using MCPB.py

Y1-M1-Y2 14.29 87.68 Created by the Seminario method using MCPB.py

Y1-M1-Y3 34.88 83.18 Created by the Seminario method using MCPB.py

Y1-M1-Y4 21.03 87.31 Created by the Seminario method using MCPB.py

Y2-M1-Y3 20.70 86.45 Created by the Seminario method using MCPB.py

Y2-M1-Y4 8.34 131.81 Created by the Seminario method using MCPB.py

Y3-M1-Y4 25.47 140.16 Created by the Seminario method using MCPB.py

2C-CO-Y3 70.0 117.00

CX-C -Y1 80.0 120.40 (was CT-C-O)

CX-C -Y2 80.0 120.40 (was CT-C-O)

CX-C -Y4 80.0 120.40 (was CT-C-O)

Y1-C -N 80.0 122.90 AA general

Y2-C -N 80.0 122.90 AA general

Y3-CO-O2 80.0 126.00

Y4-C -N 80.0 122.90 AA general

**DIHE**

2C-CO-Y3-M1 3 0.00 0.00 3.0 Treat as zero by MCPB.py

C -Y1-M1-Y2 3 0.00 0.00 3.0 Treat as zero by MCPB.py

C -Y1-M1-Y3 3 0.00 0.00 3.0 Treat as zero by MCPB.py

C -Y1-M1-Y4 3 0.00 0.00 3.0 Treat as zero by MCPB.py

C -Y2-M1-Y3 3 0.00 0.00 3.0 Treat as zero by MCPB.py

C -Y2-M1-Y4 3 0.00 0.00 3.0 Treat as zero by MCPB.py

CO-Y3-M1-Y4 3 0.00 0.00 3.0 Treat as zero by MCPB.py

CX-2C-CO-Y3 1 0.031 180.0 -4.0

CX-2C-CO-Y3 1 0.0 0.0 -3.0

CX-2C-CO-Y3 1 0.769 180.0 -2.0

CX-2C-CO-Y3 1 0.0 0.0 1.0

CX-C -Y1-M1 3 0.00 0.00 3.0 Treat as zero by MCPB.py

CX-C -Y2-M1 3 0.00 0.00 3.0 Treat as zero by MCPB.py

CX-C -Y4-M1 3 0.00 0.00 3.0 Treat as zero by MCPB.py

M1-Y1-C -N 3 0.00 0.00 3.0 Treat as zero by MCPB.py

M1-Y2-C -N 3 0.00 0.00 3.0 Treat as zero by MCPB.py

M1-Y3-CO-O2 3 0.00 0.00 3.0 Treat as zero by MCPB.py

M1-Y4-C -N 3 0.00 0.00 3.0 Treat as zero by MCPB.py

Y1-C -CX-H1 1 0.8 0.0 -1.0 Junmei et al, 1999 (was H1-CT-C -O )

Y1-C -CX-H1 1 0.0 0.0 -2.0 Explicit of wild card X-C-CT-X

Y1-C -CX-H1 1 0.08 180.0 3.0 Junmei et al, 1999 (was H1-CT-C -O )

Y1-M1-Y2-C 3 0.00 0.00 3.0 Treat as zero by MCPB.py

Y1-M1-Y3-CO 3 0.00 0.00 3.0 Treat as zero by MCPB.py

Y1-M1-Y4-C 3 0.00 0.00 3.0 Treat as zero by MCPB.py

Y2-C -CX-H1 1 0.8 0.0 -1.0 Junmei et al, 1999 (was H1-CT-C -O )

Y2-C -CX-H1 1 0.0 0.0 -2.0 Explicit of wild card X-C-CT-X

Y2-C -CX-H1 1 0.08 180.0 3.0 Junmei et al, 1999 (was H1-CT-C -O )

Y2-M1-Y3-CO 3 0.00 0.00 3.0 Treat as zero by MCPB.py

Y2-M1-Y4-C 3 0.00 0.00 3.0 Treat as zero by MCPB.py

Y3-CO-2C-HC 1 0.0 0.0 2.0

Y3-M1-Y4-C 3 0.00 0.00 3.0 Treat as zero by MCPB.py

Y4-C -CX-H1 1 0.8 0.0 -1.0 Junmei et al, 1999 (was H1-CT-C -O )

Y4-C -CX-H1 1 0.0 0.0 -2.0 Explicit of wild card X-C-CT-X

Y4-C -CX-H1 1 0.08 180.0 3.0 Junmei et al, 1999 (was H1-CT-C -O )

**IMPR**

X -O2-CO-Y3 10.5 180. 2.

**NONB**

M1 1.6080 0.0833796100 IOD set for Ca2+ ion from Li et al. JCTC, 2013, 9, 2733

Y1 1.6612 0.2100 OPLS

Y2 1.6612 0.2100 OPLS

Y3 1.6612 0.2100 OPLS

Y4 1.6612 0.2100 OPLS

**Section 2: Detailed parameters of the meta-eABF simulation**

**A. The simulation system for meta-eABF.** The system for the meta-eABF simulation was a cuboid box (98.436 Å×97.755 Å×96.459 Å) containing 23999 water molecules, 69 sodium ions, 72 chloride ions, and 1 oseltamivir molecule (a total of 78153 atoms). The thickness of the water layer was 18 Å.

**B. The sampling space.** The collective variable and sampling space were set as described in the main text. The upper bound of the sampling space on the *x* axis was 22 Å, and the lower bound was -24 Å; the upper bound on the *y* axis was 18 Å, and the lower bound was -20 Å; and the upper bound on the *z* axis was 28 Å, and the lower bound was -4 Å. We set the lower walls and upper walls, and the force constants of the upper and lower bounds of each axis were 200; that is, the range of motion of oseltamivir was limited to a cuboid box with a size of 46 Å×38 Å×32 Å. The hillweight, hillwidth and bin value were 0.02 kcal/mol, 0.25 bin width and 0.25 Å^2^ (0.5 Å×0.5 Å), respectively.

**C. The explanation for the occurrence of the region with very low free energy in the upper right corner of the free energy landscape.** We notice that there is a region with very low free energy in the upper right corner of the free-energy landscape (Fig. 6a). However, oseltamivir is not found in this region in any metastable state. After analyzing the trajectories of the meta-eABF simulation and the free-binding molecular dynamics with NA monomer, we find that this is due to the difference in the restricted positions between them. The upper right corner located at the outer side of the active site of NA (Fig. 6b) is included into the motion space of oseltamivir in the meta-eABF simulation, and oseltamivir is often found to stuck in this region for a long time. However, this region is not included into the motion space of oseltamivir in the free-binding molecular dynamics simulations with NA monomer or tetramer, therefore oseltamivir cannot reach this region. In addition, NA is a tetramer under physiological conditions, making this region sheltered by adjacent NA subunit and is inaccessible for oseltamivir molecule. Therefore, the region with very low free energy in the upper right corner of the free-energy landscape is not considered in this study.

**Section 3: Analysis of the H-bond between the carboxylate group of oseltamivir and the guanidine group of R118 in the crystal structure containing oseltamivir and influenza NA.**

**Analysis of H-bonds.** The criteria we used to evaluate the hydrogen bond between the carboxylate group of oseltamivir and the guanidine group of R118 were an angle cutoff value (hydrogen atom donor and acceptor) ≤ 30° and a radius cutoff value (hydrogen atom donor and acceptor) ≤ 3.5 Å, specifically R118:HH11-R118:NH1-G39:O1B≤30° and G39:O1B – R118:NH1 ≤3.5 Å or R118:HH21-R118:NH2-G39:O1B≤30° and G39:O1B – R118:NH2 ≤3.5 Å.

We analyzed all the crystal structures of influenza virus NA cocrystallized with oseltamivir in RCSB, including wild-type and mutant NA (Table B). There was no water-mediated hydrogen bond between the carboxylate group of oseltamivir and the guanidine group of R118 in any of the influenza virus NA-oseltamivir crystals. Although according to the above criteria for hydrogen bonding, the carboxylate group of oseltamivir and the guanidine group of R118 in the 4CPM, 2HT7 and 2HU4 crystal structures did not form a hydrogen bond, the two groups in these subunits were still very close, but water molecules could not penetrate to form a water-mediated hydrogen bond. In all the remaining crystal structures, the carboxylate group of oseltamivir and the guanidine group of R118 formed direct hydrogen bonds.

**Table B.** Basic information about the oseltamivir-NA crystal structure

| **PDB ID** | **Virus type** | **NA subtype** | **Resolution (Å)** | **Mutations** | **Distance (Å)^a^** | **Angle (°)^b^** | **Ref.** | |
| --- | --- | --- | --- | --- | --- | --- | --- | --- |
| 6HEB | A | N9 | 1.75 Å |  | 2.86 | 8.683 | | To be published |
| 6HG5 | A | N6 | 1.60 Å |  | Chain A: 2.91  Chain B: 2.96  Chain C: 2.91  Chain D: 2.87 | Chain A: 7.035  Chain B: 7.851  Chain C: 7.582  Chain D: 6.746 | | To be published |
| 5NWE | A | N1 | 2.00 Å | H275Y | Chain A: 2.87  Chain B: 2.83  Chain C: 2.93  Chain D: 2.81 | Chain A: 1.786  Chain B: 1.480  Chain C: 1.813  Chain D: 1.763 | | [1] |
| 5NZ4 | A | N1 | 1.36 Å | I223V | Chain A: 2.97  Chain B: 2.93 | Chain A: 3.805  Chain B: 6.394 | | [1] |
| 5NZN | A | N1 | 1.73 Å | H275Y,  S247N | Chain A: 2.80  Chain B: 2.89  Chain C: 2.77  Chain D: 2.88 | Chain A: 4.199  Chain B: 6.201  Chain C: 6.226  Chain D: 5.755 | | [1] |
| 5NZF | A | N1 | 1.75 Å | H275Y,  I223V | Chain A: 2.87  Chain B: 2.93  Chain C: 2.82  Chain D: 2.94 | Chain A: 1.794  Chain B: 6.225  Chain C: 5.891  Chain D: 5.434 | | [1] |
| 5NZE | A | N1 | 1.69 Å | S247N | Chain A: 2.92  Chain B: 2.94 | Chain A: 4.280  Chain B: 5.616 | | [1] |
| 5L15 | A | N9 | 2.40 Å |  | 2.87 | 4.549 | | [2] |
| 4WA4 | A | N8 | 1.95 Å |  | 2.78 | 9.458 | | [3] |
| 4QN7 | A | N7 | 2.30 Å |  | Chain A: 2.98  Chain B: 2.89 | Chain A:11.005  Chain B: 9.301 | | [4] |
| 4CPY | B |  | 1.80 Å | I221L | Chain A: 2.97  Chain B: 2.88 | Chain A: 10.920  Chain B: 9.307 | | [5] |
| 4CPM | B |  | 2.75 Å |  | Chain A: 2.59  Chain B: 2.94 | Chain A: 58.629  Chain B: 14.066 | | [5] |
| 4MWW | A | N9 | 1.90 Å | R294K,  D84N | 2.76 | 12.743 | | [6] |
| 4MWQ | A | N9 | 2.00 Å |  | 2.83 | 12.659 | | [6] |
| 4HZX | A | N3 | 2.20 Å |  | 3.13 | 15.728 | | [7] |
| 4HZZ | A | N3 | 1.60 Å | H274Y | 2.99 | 8.586 | | [7] |
| 4K1J | A | N2 | 2.20 Å | D147G | Chain A: 2.87  Chain B: 2.96 | Chain A: 5.194  Chain B: 6.408 | | [8] |
| 4K1K | A | N2 | 1.60 Å |  | Chain A: 2.85  Chain B: 2.90 | Chain A: 8.156  Chain B: 9.128 | | [8] |
| 4K1I | A | N2 | 1.80 Å |  | Chain A: 3.12  Chain B: 3.10 | Chain A: 7.181  Chain B: 5.012 | | [8] |
| 4GZP | A | N2 | 2.30 Å |  | 3.32 | 13.942 | | [9] |
| 4GZT | A | N2 | 2.19 Å | D151G | Chain A: 3.14  Chain B: 3.13  Chain C: 2.98  Chain D: 3.03 | Chain A: 13.911  Chain B: 7.681  Chain C: 3.787  Chain D: 12.672 | | [9] |
| 4B7R | A | N1 | 1.90 Å | Y351F | Chain A: 2.86  Chain B: 2.90  Chain C: 2.87  Chain D: 2.89 | Chain A: 9.082  Chain B: 4.165  Chain C: 8.133  Chain D: 6.075 | | [10] |
| 4B7J | A | N1 | 2.42 Å |  | 2.95 | 6.908 | | [10] |
| 3TI6 | A | N1 | 1.69 Å |  | Chain A: 2.84  Chain B: 2.86 | Chain A: 4.734  Chain B: 6.387 | | [11] |
| 3K3A | B |  | 2.59 Å | D197E | Chain A: 2.99  Chain B: 3.08  Chain C: 2.99  Chain D: 3.29  Chain E: 3.23  Chain F: 3.19  Chain G: 3.50  Chain H: 3.18  Chain I: 3.41  Chain J: 3.31  Chain K: 3.41  Chain L: 3.14  Chain M:3.16  Chain N: 2.98  Chain O: 3.22  Chain P: 3.09 | Chain A: 15.906  Chain B: 16.321  Chain C: 12.172  Chain D: 18.522  Chain E: 20.443  Chain F: 21.379  Chain G: 17.427  Chain H: 16.582  Chain I: 18.128  Chain J: 16.914  Chain K: 15.274  Chain L: 13.827  Chain M: 17.494  Chain N: 13.923  Chain O: 17.699  Chain P: 11.893 | | [12] |
| 3CL0 | A | N1 | 2.20 Å | H274Y | 2.70 | 9.579 | | [13] |
| 3CL2 | A | N1 | 2.54 Å | N294S | Chain A: 3.29  Chain B: 3.17  Chain C: 3.23  Chain D: 3.08  Chain E: 3.27  Chain F: 3.20  Chain G: 3.07  Chain H: 3.16 | Chain A: 12.037  Chain B: 14.649  Chain C: 20.255  Chain D: 15.012  Chain E: 15.467  Chain F: 12.019  Chain G: 18.083  Chain H: 15.607 | | [13] |
| 2HT8 | A | N8 | 2.40 Å |  | 3.18 | 16.293 | | [14] |
| 2HT7 | A | N8 | 2.60 Å |  | 3.52 | 18.889 | | [14] |
| 2HU4 | A | N1 | 2.50 Å | H252Y | Chain A: 3.62  Chain B: 3.41  Chain C: 3.39  Chain D: 3.48  Chain E: 3.49  Chain F: 3.43  Chain G: 3.56  Chain H: 3.82 | Chain A: 16.823  Chain B: 7.598  Chain C: 8.267  Chain D: 16.711  Chain E: 12.198  Chain F: 10.266  Chain G: 19.149  Chain H: 15.712 | | [14] |
| 2HU0 | A | N1 | 2.95 Å | H252Y | 3.44 | 7.602 | | [14] |
| 2QWK | A | N9 | 1.80 Å |  | 2.81 | 8.523 | | [15] |
| 2QWH | A | N9 | 1.80 Å | R292K | 2.77 | 12.727 | | [15] |

① PROTOSS was used to add hydrogen atoms to all crystal structures and to analyze the H-bonds [16, 17].

② G39 was the residue name in the crystal structures.

③ The distance and angles of atom pairs between oseltamivir and R118 were analyzed for each subunit of every crystal structure using VMD [18].

^a^ G39:O1B -R118:NH1 or G39:O1B – R118:NH2.

^b^R118:HH11-R118:NH1-G39:O1B or R118:HH21-R118:NH2-G39:O1B.

**Section 4: Transition path theory fluxes from the unbound state (S1) to the bound state (S9).**

**Table C.** Net flux (×10^-6^/ns) from the unbound state (S1) to the bound state (S9).

| Final state  Initial state | S0 | S1 | S2 | S3 | S4 | S5 | S6 | S7 | S8 | S9 |
| --- | --- | --- | --- | --- | --- | --- | --- | --- | --- | --- |
| S0 | 0 | 0 | 2.168 | 0.414 | 1.141 | 0 | 0.551 | 1.161 | 0 | 0 |
| S1 | 5.434 | 0 | 4.142 | 0 | 0.368 | 0.059 | 11.802 | 6.388 | 0.247 | 0 |
| S2 | 0 | 0 | 0 | 0.317 | 0 | 0 | 0 | 10.043 | 0 | 0.164 |
| S3 | 0 | 0 | 0 | 0 | 0 | 0 | 0 | 0 | 0 | 13.693 |
| S4 | 0 | 0 | 1.941 | 0 | 0 | 0 | 0 | 0.264 | 0 | 0 |
| S5 | 0 | 0 | 0 | 0 | 0 | 0 | 0.059 | 0 | 0 | 0 |
| S6 | 0 | 0 | 2.272 | 4.782 | 0.697 | 0 | 0 | 4.661 | 0 | 0 |
| S7 | 0 | 0 | 0 | 8.031 | 0 | 0 | 0 | 0 | 4.868 | 9.617 |
| S8 | 0 | 0 | 0 | 0.149 | 0 | 0 | 0 | 0 | 0 | 4.966 |
| S9 | 0 | 0 | 0 | 0 | 0 | 0 | 0 | 0 | 0 | 0 |

**Section 5: Pathway analysis based on metastable states**

**Table D.** Dominant reaction pathways between the unbound state (S1) and the bound state (S9)

| Pathway | Path flux (×10^-6^/ns) | Cumulative path flux (×10^-6^)/ns | Percentage of total path flux (%) |
| --- | --- | --- | --- |
| [1, 7, 9] | 6.388 | 6.388 | 22.46 |
| [1, 6, 3, 9] | 4.782 | 11.171 | 16.82 |
| [1, 6, 7, 3, 9] | 4.661 | 15.832 | 16.39 |
| [1, 2, 7, 8, 9] | 4.142 | 19.974 | 14.57 |
| [1, 6, 2, 7, 3, 9] | 2.272 | 22.246 | 7.99 |
| [1, 0, 2, 7, 9] | 2.168 | 24.414 | 7.62 |
| [1, 0, 7, 3, 9] | 1.098 | 25.513 | 3.86 |
| [1, 0, 4, 2, 7, 9] | 1.061 | 26.573 | 3.73 |
| [1, 0, 3, 9] | 0.414 | 26.987 | 1.46 |
| [1, 0, 6, 4, 2, 7, 8, 9] | 0.399 | 27.387 | 1.4 |
| [1, 4, 2, 3, 9] | 0.317 | 27.703 | 1.11 |
| [1, 8, 9] | 0.247 | 27.950 | 0.87 |
| [1, 0, 6, 4, 7, 8, 9] | 0.152 | 28.102 | 0.53 |
| [1, 6, 4, 2, 9] | 0.086 | 28.188 | 0.3 |
| [1, 0, 4, 7, 8, 3, 9] | 0.080 | 28.268 | 0.28 |
| [1, 0, 7, 8, 3, 9] | 0.062 | 28.330 | 0.22 |
| [1, 5, 6, 4, 2, 9] | 0.059 | 28.389 | 0.21 |
| [1, 4, 7, 8, 9] | 0.026 | 28.415 | 0.09 |
| [1, 4, 2, 9] | 0.018 | 28.433 | 0.06 |
| [1, 4, 7, 8, 3, 9] | 0.006 | 28.439 | 0.02 |

Pathways with fluxes less than 0.61×10^-8^/ns were excluded when calculating the cumulative path flux and percentage of the total path flux.

**Section 6: Mean first passage time between metastable states**

**Table E.** Mean first passage time between metastable states.

| **Initial state i** | **Final State j** | **Time (ns)** |
| --- | --- | --- |
| 1  7  1  6  3  6  7  1  2  7  8  6  1  0  0  0  4  0  0  6  1  2  1  2  4  8  1  5  1 | 7  9  6  3  9  7  3  2  7  8  9  2  0  2  7  4  2  3  6  4  4  3  8  9  7  3  5  6  9 | 512  415  8517  527  393  586  496  6371  168  726  724  6841  17362  5968  506  51458  2246  582  10779  54096  53461  532  887  469  342  736  77993  8422  577 |

**Section 7:** [**Comparison**](https://cn.bing.com/dict/search?q=comparisons&FORM=BDVSP6&cc=cn) [**of**](https://cn.bing.com/dict/search?q=of&FORM=BDVSP6&cc=cn)**residue numbers of NA between our simulation system and the PDB 2QWK crystal structure**

**Table F.** [Comparison](https://cn.bing.com/dict/search?q=comparisons&FORM=BDVSP6&cc=cn) [of](https://cn.bing.com/dict/search?q=of&FORM=BDVSP6&cc=cn) residue numbers of NA between our simulation system and the crystal structure PDB 2QWK

| **Regions** | **Residue number in crystal structure 2QWK** | **Residue number in the simulations with an NA monomer** |
| --- | --- | --- |
| 150 loop region | 147–152 | 66–71 |
| 370 loop region | 366–373 | 285–292 |
| 400 loop region | 399–404 | 317–322 |
| 430 loop region | 429–433 | 349–353 |
| 250 loop region | 244–250 | 164–170 |
| 270 loop region | 268–275 | 188–195 |
| Ca^2+^ and coordinated residues | Ca^2+^: 999, coordinated residues: D293, G297, D324, and N347 | Ca^2+^: 389, coordinated residues: D213, G217, D244, and N266 |
| the 1° site | R118, E119, D151, R152, R292, and R371 | R37, E38, D70, R71, R212, and R290 |
| the 2° site | S367, I368, A369, S370, S372, N400, W403, and K432 | S286, I287, A288, S289, S291, N318, W321, and K352 |
| metastable state S0B | P326, R327, G343, N344, N345, N346, N347, G348, and A369 | P246, R247, G262, N263, N264, N265, N266, G267, and A288 |
| metastable state S0C | R118, I149, D151, R430, and P431 | R37, I68, D70, R350, and P351 |
| metastable state S5 | A250, E251, P249, Lys273, A272, I275, and G248 | A170, E171, P169, Lys193, A192, I195, and G168 |
| metastable state S6 | R152, S153, Gln154, and N198 | R71, S72, Gln73, and N118 |
| metastable state S2B | R371, W403, and A369 | R290, W321, and A288 |
| metastable state S2A | R371, R292, P431, and I427 | R290, R212, P351, and I347 |
| metastable state S3 | R152, D151, E119, R224, and W178 | R71, D70, E38, R144, and W98 |
| metastable state S9 | R152, R371, R292, D151, E119, W178, I222, A246, E276, E277, N294, and R118 | R71, R290, R212, D70, E38, W98, I142, A166, E196, E197, N214, and R37 |
| metastable state S4 | R118, R371, R292, D151, W403, and R430 | R37, R290, R212, D70, W321, and R350 |
| metastable state S7 | R371, R292, D151, A246, and N294 | R290, R212, D70, A166, and N214 |

**References:**

1. Pokorna J, Pachl P, Karlukova E, Hejdanek J, Rezacova P, Machara A, et al. Kinetic, Thermodynamic, and Structural Analysis of Drug Resistance Mutations in Neuraminidase from the 2009 Pandemic Influenza Virus. Viruses. 2018;10(7):339.
2. Gubareva LV, Sleeman K, Guo Z, Yang H, Hodges E, Davis CT, et al. Drug Susceptibility Evaluation of an Influenza A(H7N9) Virus by Analyzing Recombinant Neuraminidase Proteins. J Infect Dis. 2017;216:S566-S574.
3. Yang H, Nguyen HT, Carney PJ, Guo Z, Chang JC, Jones J, et al. Structural and functional analysis of surface proteins from an A(H3N8) influenza virus isolated from New England harbor seals. J Virol. 2015;89(5):2801-2812.
4. Sun X, Li Q, Wu Y, Wang M, Liu Y, Qi J, et al. Structure of influenza virus N7: the last piece of the neuraminidase "jigsaw" puzzle. J Virol. 2014;88(16):9197-9207.
5. Escuret V, Collins PJ, Casalegno JS, Vachieri SG, Cattle N, Ferraris O, et al. A novel I221L substitution in neuraminidase confers high-level resistance to oseltamivir in influenza B viruses. J Infect Dis. 2014;210(8):1260-1269.
6. Wu Y, Bi Y, Vavricka CJ, Sun X, Zhang Y, Gao F, et al. Characterization of two distinct neuraminidases from avian-origin human-infecting H7N9 influenza viruses. Cell Res. 2013;23(12):1347-1355.
7. Li Q, Qi J, Wu Y, Kiyota H, Tanaka K, Suhara Y, et al. Functional and structural analysis of influenza virus neuraminidase N3 offers further insight into the mechanisms of oseltamivir resistance. J Virol. 2013;87(18):10016-10024.
8. Wu Y, Qin G, Gao F, Liu Y, Vavricka CJ, Qi J, et al. Induced opening of influenza virus neuraminidase N2 150-loop suggests an important role in inhibitor binding. Sci Rep. 2013;3:1551.
9. Zhu X, McBride R, Nycholat CM, Yu W, Paulson JC, Wilson IA. Influenza virus neuraminidases with reduced enzymatic activity that avidly bind sialic Acid receptors. J Virol. 2012;86(24):13371-13383.
10. van der Vries E, Collins PJ, Vachieri SG, Xiong X, Liu J, Walker PA, et al. H1N1 2009 pandemic influenza virus: resistance of the I223R neuraminidase mutant explained by kinetic and structural analysis. PLoS Pathog. 2012;8(9):e1002914.
11. Vavricka CJ, Li Q, Wu Y, Qi J, Wang M, Liu Y, et al. Structural and functional analysis of laninamivir and its octanoate prodrug reveals group specific mechanisms for influenza NA inhibition. PLoS Pathog. 2011;7(10):e1002249.
12. Oakley AJ, Barrett S, Peat TS, Newman J, Streltsov VA, Waddington L, et al. Structural and functional basis of resistance to neuraminidase inhibitors of influenza B viruses. J Med Chem. 2010;53(17):6421-6431.
13. Collins PJ, Haire LF, Lin YP, Liu J, Russell RJ, Walker PA, et al. Crystal structures of oseltamivir-resistant influenza virus neuraminidase mutants. Nature. 2008;453(7199):1258-1261.
14. Russell RJ, Haire LF, Stevens DJ, Collins PJ, Lin YP, Blackburn GM, et al. The structure of H5N1 avian influenza neuraminidase suggests new opportunities for drug design. Nature. 2006;443(7107):45-49.
15. Varghese JN, Smith PW, Sollis SL, Blick TJ, Sahasrabudhe A, McKimm-Breschkin JL, et al. Drug design against a shifting target: a structural basis for resistance to inhibitors in a variant of influenza virus neuraminidase. Structure.
16. Lippert T, Rarey M. Fast automated placement of polar hydrogen atoms in protein-ligand complexes. J Cheminform. 2009;1(1):13.
17. Bietz S, Urbaczek S, Schulz B, Rarey M. Protoss: a holistic approach to predict tautomers and protonation states in protein-ligand complexes. J Cheminform. 2014;6:12.
18. Humphrey W, Dalke A, Schulten K. VMD: visual molecular dynamics. J Mol Graph. 1996;14(1):33-28.
