# Supplementary material for: Binding mechanism of oseltamivir and influenza neuraminidase suggests perspectives for the design of new anti-influenza drugs
Source: PLoS Comput Biol. 2022 Jul 28;18(7):e1010343. doi: 10.1371/journal.pcbi.1010343 (PMC9401145; doi:10.1371/journal.pcbi.1010343)
Supplement: Attachment — Submitted filename: responses to the review comments.doc [file pcbi.1010343.s015.doc]

**Response the comments of the reviewers**

**Response the comments of Reviewer #1:**

1. ① Although 10 meta-stable states were identified using 'constrained' free (?) MD using ligand at a distance from the binding, ② it would have been appropriate to show the stability of protein-ligand complex in MD starting from the crystal structure and whether a water-mediate H-bond between ligand and Arg118 is observed in MD using the bound (crystal) state. This could have served as an additional validation.

**Response：**

**①** To save the computational resource, in our free-binding simulation, the motion range of oseltamivir was limited to a hemispherical region with a radius of 27 Å above the 1° site (Fig 1a). In such simulation, oseltamivir molecule can interact freely with the 1° and 2° sites. When the distance between oseltamivir molecule and the active center of NA is over 27 Å, a restraint will be applied to pull oseltamivir molecule toward the active center of NA to avoid the oseltamivir molecule diffusing away（please see the response to question 3 of reviewer 2 for detailed method for this simulation）.

**②** In the past three weeks, we followed the reviewer’s advice to investigate the stability of protein-ligand complex in MD starting from the crystal structure and whether a water-mediate H-bond between the ligand and Arg118 is observed in MD using the bound (crystal) state.

We performed four separate 12-μs simulations with N9 type NA tetramer (The NA tetramer was prepared by matrix transformation starting from the 2QWK structure as described in the section of **Materials and Methods**.), resulting a total of 16 MD trajectories for individual oseltamivir-NA complex.

The RMSD referring to the crystal structure (2QWK) of oseltamivir and NA protein were calculated. We found that all the RMSD values of oseltamivir in the simulations varied between 0.15 and 1.8 Å, and in 10 trajectories the RMSD values of oseltamivir varied between 0.15 and 0.8 Å (a fluctuation of 0.65 Å) all through the simulations, indicating a considerably stable binding state of oseltamivir in the active centor of NA. The RMSD of NA varied between 0.7 and 2.4 Å (a fluctuation of 1.7 Å).

The distance (DON) between the O1 atom of oseltamivir and NH1 atom of Arg118 in each oseltamivir-NA complex was plotted (S5 Fig, please see below), and the distance values of each trajectory and all the 16 trajectories was calculated. In 15 trajectories (93.3%), the survival time of the direct H-bond was less than 10 ns (less than 0.83% simulation time), and most of them only existed at the beginning of the simulation. Only in 1 trajectory, the direct H-bond persisted for about 550 ns at the initial stage of the simulation but disappeared later. The average DON of individual oseltamivir-NA complex were between 5.27 Å and 6.84 Å, and the average distance value of all oseltamivir-NA complex is 5.96Å. By manually analyzing the relationship between the water-mediated H-bond and the DON, we found that the most favorable distance for hydrogen bond formation is about 6Å (data not shown). So, we think that there is stable water-mediated H-bond between oseltamivir and NA in the stable binding state in our simulations. We have uploaded all the MD files (S1 data) for the simulations.

**S5 Fig. The stability of oseltamivir-NA complex and water-mediated H-bond.** The simulations were performed with osertamivir-NA complex of NA tetramer. (a) The RMSD valuse of oseltamivir and (b) NA. (c) RMSD of the 16 individual oseltamivir-NA complex of the . (d) Column 1-16 is the average DON of individual oseltamivir-NA complex (5.27 Å ~ 6.84 Å), column 17 is the average DON of all oseltamivir-NA complex (5.96Å).

2. It would be difficult to isolate the effect of the force field (and simulation parameters) for the Metastable states S3, S4, S8 and S9 where Oseltamivir adopts alternate bound configurations compared to the one observed in the crystal structure. It has been challenging to obtain a stable protein-ligand complex between Neuraminidase (and similar viral proteins with 3 Arg in the binding site) and ligand in long MD simulations as observed from published (10.26434/chemrxiv-2021-56ds7), especially for monomers (compared to tetramer for neuraminidase).

**Responses：**In fact, before the massive MD, we were not sure about the validity the force field and charge parameters of oseltamivir (GAFF) and protein (ff14SB) adopted in this study. **①** So we carried out tentative 10 simulations (1200 ns each) to find whether oseltamivir molecule can reach and keep stable binding state as that of crystal structure. We found that 3 simulations (2 within 600 ns and 1 beyond 600 ns) produced positive binding events (data not shown). Once oseltamivir bound to the 1° site with a RMSD of less than 2 Å *vs*. that of the crystal structure, it remained steady and never leave the 1° site till the end of the simulation (please see Figure 1). **②** We also performed further 600-ns simulations started from the final conformation of the positive simulations, and found that oseltamivir molecule can still keep the same stable binding state as the starting conformation. **③** We performed the binding-state simulations for zanamivir and peramivir with force field and charge parameters prepared using the same method as oseltamivir, and found the two molecules can also kept stable states as oseltamivir did . **④** As described in the above response, we performed long-time (1200 μs) simulations started from the tetramer co-crystal structure of oseltamivir and NA. The results showed that the four oseltamivir-NA complex in the tetramer kept the same stable binding state as observed in free-binding simulations.

In conclusion, the force field and charge parameters of oseltamivir and protein (ff14SB) adopted in this study produced stable protein-ligand conformation in either free-binding simulation or binding state simulation, which suggested the validity of the combination of the force field, charge parameters and simulation parameters. Since we have obtained the final stable binding state in the massive free binding simulations as that of the crystal structure, we speculated that the metastable states S3, S4, S8 and S9 in this study should ha.

We had read the paper (DOI: 10.26434/chemrxiv-2021-56ds7) recommended by the reviewer, published in 2021. The major differences between this and our study is that this study used a relatively older force field, AMBER99SB-ILDN force field, while we used a newer force field, AMBER ff14SB and AMBER 18. As we know the force field of protein has been on the way of optimization, and newer force field parameter may provide more ideal simulation result. We speculate that it may be the difference in the protein force field caused the difference in the stability of oseltamivir between that study and ours.

3. It would be good to see the stability of the water-mediated complex between NA (Arg118) and Oseltamivir in longer MD simulations.

**Responses：**Please refer to the response to the first question for detailed information.

4. Additional findings suggest that metastable state S4, the ligand adopts an alternate bound conformation compared to the crystal structure state. Thermodynamic stability/preference between these two states (not given in the paper) would be useful to choose one or both states for drug design.

**Responses：**S4 is an intermediate state during the transition of oseltamivir to S9 (stable binding state). **By manually analyzing the trajectories, we found that the duration of S4 is usually 10 ns to 100 ns (data not shown)**. However, we found it’s impossible to mark the duration of S4 in a RMSD figure due the overlap of RMSD lines. So we can only provide the description for stability of S4 (the bold words). This data will be added to the revised manuscript. Though the stability of S4 is far less stable than that of S9, our MSM results show that S4 usually transit to S2 and S7. Especially, the MFPT of S4 to S2 is 2246 ns (please see Table S3, Table S4, Table S5 in the S1 Text), which indicates that the transition speed of S4 to the S2 is slow. Therefore, we speculate that it is possible to design new drugs based on S4.

5. Other Meta-stable states and binding pathways have less bearing on the design of better inhibitors.

**Responses：**Yes, we also think that the other meta-stable states and binding pathways have less bearing on the design of better inhibitors, except metastable state S4 and S9, so we did not give any disscussion on them.

**Response to the comments of reviewer #2:**

In this study, the authors perform a large number of molecular dynamics (MD) simulations of neuraminidase (NA) monomers and tetramers with the oseltamivir inhibitor for the purpose of performing a Markov state model (MSM) analysis of various binding and metastable states. The authors also perform a number of free energy and kinetics calculations, and suggest some chemical modifications to existing NA inhibitors that, the authors allege, may enable the production of new anti-influenza drugs. The authors identify nine states, which define the unbound state, the active site and secondary site bound states, and a number of other metastable states, all of which interact with certain key residues and loops that have (and on occasion have not) been previously identified in the literature. Significantly, the authors find that a relatively high free energy barrier exists between the active site and the secondary site of NA for oseltamivir, opposing the hypothesis that the secondary site acts as a sort of bridge from the solvent to the active site. In addition, the authors focus on the large gap, likely a water-mediated hydrogen bond, between existing inhibitors, including oseltamivir, and residue R118. The authors believe that this gap represents an ideal drug design opportunity.

This reviewer believes that the authors have performed a significant and meaningful study of the binding mechanisms between NA and oseltamivir, and that this manuscript contains valuable and interesting information for the broader scientific community. However, minor revisions are suggested before acceptance for publication, and the manuscript has a number of weaknesses, especially spelling and grammar mistakes, which undermine what would otherwise seem to be a valid study.

Minor Revisions:

1. The first sentence of the manuscript on line 65 of page 4 “Among influenza viruses, influenza A is the most harmful” is unsupported by references or further information. It is also unclear how this statement fits with the rest of the study (is the study performed on an influenza A neuraminidase?).

**Response：**We have replaced this sentence with “Influenza A virus has been a serious health concern to humanity, with high morbidity and mortality rates in human history [1-3]”. Yes, this study performed on the influenza A neuraminidase.

2. On line 82 of page 4, “crystal structures of oseltamivir-NA have been resolved” should be clearer by saying “crystal structures of oseltamivir-NA holo complexes have been resolved”.

**Response：**We have adopted the phrase suggested by Prof. Amaro in the revised manuscript.

3. The authors use the “position restraint method” to keep the ligand within 27 Angstroms of the active site of NA. What is this method? Is this a harmonic function or step function in the potential energy? If this is a force, what is its mathematical form? If it is not a force, then how exactly does this work?

**Response：**The “position restraint method” derives from the NMR refinement function of AMBER. When the distance between the beta carbon atom of N346 and the C5 atom of oseltamivir is beyond 27 Å , a penalty potential of 1 kcal·mol^-1^·Å^-2^ is applied to prevent oseltamivir molecules from escaping from the limited region.

The restraint is a well with a square bottom between r2 and r3, with parabolic sides out to a defined distance (r1 and r4 for lower and upper bounds, respectively), and then linear sides beyond that (This is shown in the following plot). If R is the value of the restraint in question:

• R < r1 Linear, with the slope of the "left-hand" parabola at the point R=r1.

• r1 <= R < r2 Parabolic, with restraint energy k2(R−r2)^2^.

• r2 <= R < r3 E = 0.

• r3 <= R < r4 Parabolic, with restraint energy k3(R−r3)^2.^

• r4 <= R Linear, with the slope of the “right-hand” parabola at the point R=r4.

We set r1 = 1.30 Å, r2 = 1.80 Å, r3 = 27.00 Å, r4 = 27.50 Å, rk2 = 0 kcal·mol^-1^·Å^-2^, rk3 = 1.0 kcal·mol^-1^·Å^-2^. rk2 and rk3 values are the force constants for the lower and upper bounds, respectively. We set rk2 = 0 kcal·mol^-1^·Å^-2^ because the van der Waals parameters in the force field will not allow two non-bonded atoms to get closer than they should be.

This restraint method is used for the entire production simulations. Please refer to the S1_Data/NA monomer-oseltamivir system/md.in and S1_Data/NA monomer-oseltamivir system/RST files for detailed methods.

4. The authors may consider using “MD” instead of “molecular dynamics” throughout the manuscript for brevity.

**Response：**We have replaced “molecular dynamics” by “MD” in the revised manuscript.

5. In the Fig. 1 caption, the authors say, “the CB of N346”. They should explicitly say “beta carbon” instead of CB, to be clearer.

**Response：**“CB”has been replace by “beta carbon atom” all through the manuscript.

6. On line 138 of page 7, the authors say: “[the different binding times observed in the trajectories] indicate that the oseltamivir-NA binding process is complex and there may be multiple binding pathways”. This is not a valid statement. It is perfectly possible to obtain an entire distribution of observed trajectory times for a molecular process due to Brownian motion, even if there is only one pathway. The evidence for multiple pathways in this study, rather, comes from the MSM results.

**Response：**This statement has been deleted in the revised manuscript.

7. Page 8 line 154 misspelled “below”. (Lots of spelling and grammar errors, the authors should check their spelling and grammar carefully.)

**Response：**The misspelling was corrected, and we also check our manuscript for spelling and grammar errors, and made corrections in the revised manuscript. .

8. Page 10 line 186, should the authors say “dotted square” instead of “dotted line”?

**Response：**“dotted line” was replaced by “dotted square” in the revised manuscript.

9. Page 16 line 296 has an incomplete sentence.

**Response：**Yes, a word “study” was added to the end of this sentence. The complete sentence is as follows: However, this speculation has not been directly verified until this **study**.

10. If possible, the authors should add error margins to Table 1.

**Response：**The numbers in Table 1 are the lowest free energy values and isn’t average ones.

11. Page 20, line 352, the word “recurred” should probably be “repeated”.

**Response：**This word “recurred” was replaced by “repeated” in the revised manuscript.

12. Page 21, line 362, misspelled “simulations”

**Response：**This misspelling was corrected in the revised manuscript.

13. Page 21, lines 366 to 368 has a different font than the rest of the manuscript.

**Response：**The fond was changed in the revised manuscript.

14. Page 21, line 370 misspelled “destroyed”.

**Response：**The misspelling was corrected in the revised manuscript.

15. The entire “Analysis of the interaction between R118 and oseltamivir is full of more spelling and grammar errors - too many to list here.

**Response：**We have checked the whole section our for spelling and grammar errors, and made corrections in the revised manuscript.

16. Please define the meaning of “physical temperature” one page 21, line 377. Is there something significant about 37 degrees celsius?

**Response：**Here we use “physical temperature” to emphasize that the crystal structures are often prepared under a lower temperature conditions that is different from normal human body temperature, *ie*, 37℃.

17. Page 24, lines 422 to 425 have two redundant sentences that are probably saying the same thing.

# Response：We deleted the former sentence, and retain the latter one: Therefore, we believe that it is highly feasible to modify the C-3 moiety of oseltamivir to enhance its interaction with the 425 R118 of NA [17].

18. Page 25, line 441, why are the authors choosing to use MCPB.py for a calcium ion? Unless the metal is covalently bound to the protein, MCPB.py is unnecessary, and the authors can just use the ordinary calcium ion parameters. If the calcium is covalently bound, then the calcium cannot be an ion. The authors should explain why they used MCPB.py, whether the calcium is an ion, and why this choice will not damage the validity of the MD simulations.

**Response：**Before constructing the simulation system, we carefully analyzed the crystal structure of published type A neuraminidase, and found that most of the crystal structure contained calcium ions near the active center and formed coordination bonds with the amino acids. Many studies show that the Ca^2+^ ion located near the centre of the NA tetramer is a major stability determinant, as this Ca^2+^ ion is required for catalysis and its binding affinity. Therefore, we adopted MCPB.py to construct the bonded model for the Ca^2+^, so that calcium ions can stably exist in the NA structure. In our simulations, we found that the oseltamivir molecules can bind to the active center of NA and formed highly consistent conformation with that of co-crystal structure of NA and OC, so we think that this bonded model did not damage the validity of the MD simulations

19. The authors should explain better why NAMD was used for the free energy landscape, and not AMBER, as with the other simulations. It is unusual to use two separate MD simulation programs for the same study.

**Response：**This study began in 2018. At the beginning, we also planned to perform metadynamics simulations using AMBER to investigate the free-energy landscape. But we found another metadynamics method, meta-eABF, which merges the key features of metadynamics and an extended Lagrangian variant of ABF (eABF) by simultaneously shaving the barriers and flooding the valleys of the free energy landscape, and possesses a convergence rate up to 5-fold greater than those of other importance-sampling algorithms (J. Phys. Chem. Lett. 2018, 9, 16, 4738–4745).

The developers of meta-eABF first introduced the method of performing meta-eABF simulations in NAMD. Considering compatibility issues, we adopted NAMD for meta-eABF simulation instead of AMBER at the time.

Though we used AMBER in massive free-binding MD simulations, we finally found that the results of metastable state derived from massive MD simulation matched well the free energy landscape derived from NAMD.
